# Effects of center pivot sprinkler fertigation on the yield of continuously cropped soybean

**DOI:** 10.1515/biol-2020-0092

**Published:** 2020-12-31

**Authors:** Hua Cao, Yongshen Fan, Zhen Chen, Xiuqiao Huang

**Affiliations:** Farmland Irrigation Research Institute, Chinese Academy of Agricultural Sciences/Key Laboratory of Water-Saving Agriculture of Henan Province, Xinxiang, 453000, China; Graduate School of Chinese Academy of Agricultural Sciences, Beijing, 100081, China

**Keywords:** center pivot sprinkler, fertigation, microspray, soybean, continuous cropping, yield

## Abstract

Continuous cropping is a common agricultural practice in Northeast China. Focusing on soybeans cropped continuously for two consecutive years, this article fully explores the effects of the amount of water, fertilizing rate, and fertilizing method on the growth and yield of soybean. Specifically, an orthogonal experimental plan was designed involving these three factors. Each factor was divided into three levels: the amount of water was set as 52.62 mm (W1), 73.41 mm (W2), and 138.6 mm (W3); the fertilizing rate was set as 6.75 kg/hm^2^ (N1), 9.75 kg/hm^2^ (N2), and 13.5 kg/hm^2^ (N3); and the fertilizing method was set as center pivot sprinkler (CPS) fertigation (F1), microspray (MS) fertigation (F2), and MS fertilizing + CPS spraying and leaching (F3). During the experiments, the growth traits at each growth stage were monitored, and the soybean yield was measured. The following results were obtained through the analysis of the experimental data: the amount of water significantly affects the growth traits of soybean in the early stage of growth; the fertilizing rate greatly affects the stem diameter; and the fertilizing method is a major influencer of soybean yield. The highest yield (2811.88 kg/hm^2^) was observed in zone 4 (W2N1F2). This means irrigation and fertilization are very important to the normal growth of continuously cropped soybean; the yield loss induced by continuous cropping can be mitigated effectively through timely and adequate irrigation and topdressing, plus fertilization by the suitable method. To prevent yield loss, farmers in Northeast China are suggested to replace continuous cropping with crop rotation. If continuous cropping is unavoidable, foliage fertilizer should be sprayed timely for topdressing at the flowering and seed-filling stages.

## 1 Introduction

Soybean is an agricultural product consumed across the globe [1]. Besides contributing to more than one-fourth of the world’s human food and animal feed [2], the crop provides an important edible oil [3] and an attractive raw material of biodiesel [4]. As a native crop in East Asia, the wild form of soybean could be found in Northeast China and the Korean Peninsula [5]. Approximately 38% of global soybean is produced in the USA [6].

Soybean is a key crop in Northeast China, especially in Heilongjiang Province. The soybean yield in this province accounts for nearly one-third of the total yield in China [7]. Each year, only one season of crop is achievable due to the cold climate in Heilongjiang. This gives rise to the practice of continuous cropping, particularly in the cold northern region. It is estimated that one-third to half of soybeans in this province are cropped continuously.

Due to continuous cropping, soybean yield has been falling year after year. In Heilongjiang, soybean yield is reducing 11–35% each year and 60–70% in severe cases. What is worse, the soybean quality is also on the decline. For local farmers, continuous cropping has grown into a thorny problem. The most widely used measures to solve the problem include seed selection and land preparation. For example, the land for continuously cropped soybean should be prepared in autumn rather than spring, so as to reduce plant diseases and insect pests.

There are also some auxiliary measures such as rational fertilizing and biodiversity improvement [8]. The center pivot sprinkler (CPS)-based fertigation is one such auxiliary measure. The CPS and lateral moves are the key to movable irrigation systems. It is a flexible tool that can adapt to various crops, soils, and landforms [9,10]. Featuring large coverage and high automation [11], the CPS has been widely used in Northeast China [12] to facilitate fertilization. However, the large outflow and the high sprinkling intensity limit the application of the CPS in foliage fertilizing.

In our experiment, a microspray (MS) fertigation system that couples the CPS with an MS system has been designed. With a small outflow and a low intensity of droplets, the MS nozzles in the system can apply foliage fertilizer without damaging the young leaves. Meanwhile, the CPS reduces the fertilizing rate by maintaining the fertilization nonintense and frequent. In this way, the system designed adjusts the fertilization according to local conditions and changes the traditional notion that a higher fertilizing rate means better yield. After all, fertilizer-intense agriculture brings a high environmental cost.

In this study, three fertilizing methods are compared, namely, CPS fertigation, MS fertigation, and MS fertilizing + CPS spraying and leaching. Note that MS fertigation is realized by integrating an MS system into a CPM. The MS system is independent of the CPM. The experiments were carried out on a soybean field that has been cropped continuously for two consecutive years (2017 and 2018). The effect of each method was tested with three amounts of water and three fertilizing rates.

## 2 Materials and methods

### 2.1 Experimental area

The experimental area (altitude: 152 m) is located in Heilongjiang Hydraulic Experiment and Research Center (N: 45°38′36″; E: 126°22′38″) and belongs to the temperate continental monsoon climate zone. The mean annual rainfall is 569.1 mm. The rainy season lasts from June to September.

The experimental area is dominated by silty loam. In the topsoil, the mean content of the organic matter is 2.38%, and the mean pH is 8.32. The other parameters of the topsoil are as follows: the mean mass ratios of nitrate, total nitrogen, total phosphorus, alkali-hydrolyzable nitrogen, effective phosphorus, and available potassium are 15.02 mg/kg, 1.52 g/kg, 0.538g/kg, 17.294 g/kg, 130.09 mg/kg, 4.48 mg/kg, and 159.50 mg/kg, respectively.

### 2.2 Materials

Our experiments were conducted with an intelligent remote-controlled CPS produced by Lindsay. The full length of the 3-span CPS is 90 m, including 40.5 m in the first span, 40.5 m in the second span, and 9 m in the third span. In the third span, there is a spray gun with a sprinkling width of 25 m. Overall, the CPS has an irrigation radius of 115 m. The sprinklers are 1.5 m above the ground on the 3.5 m-tall CPS.

**Figure 1:**
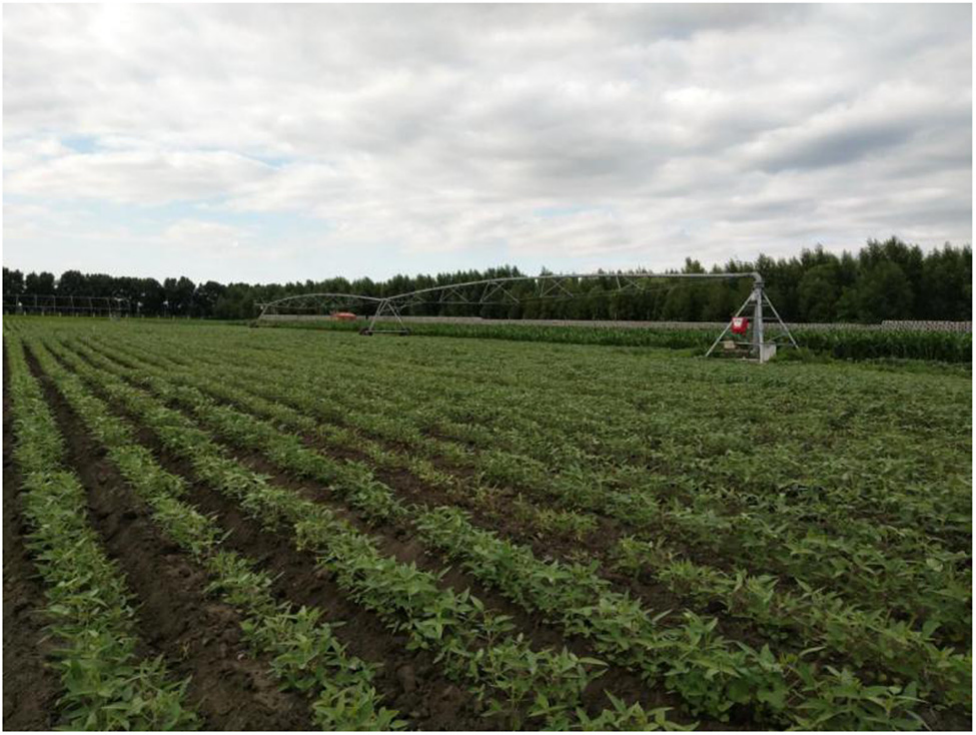
Photograph of the original CPS.

**Figure 2:**
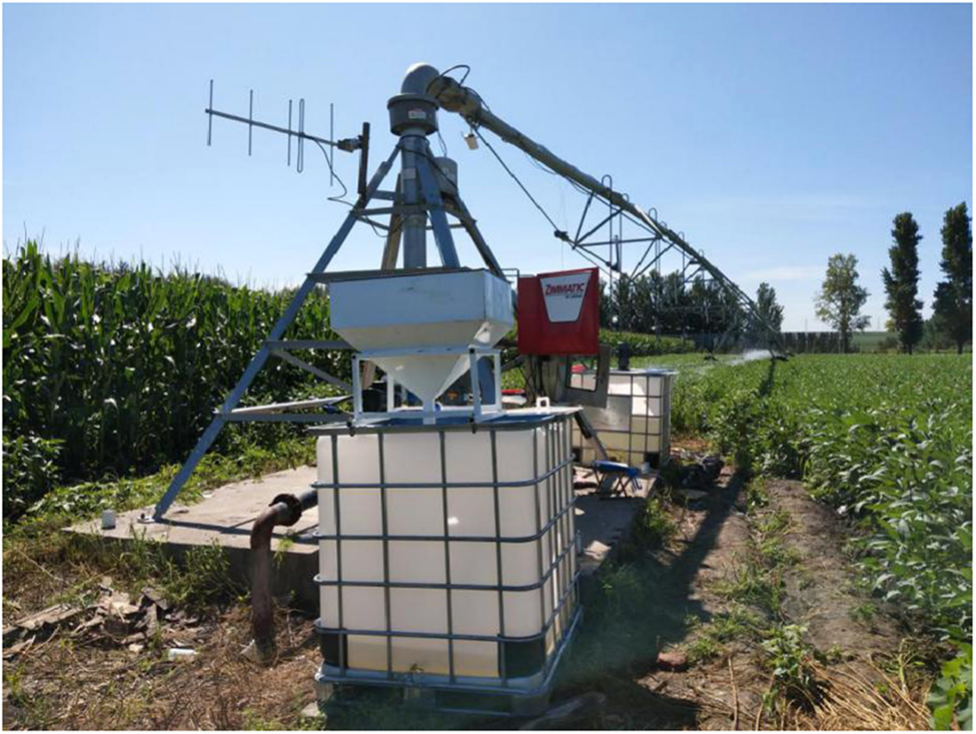
Photograph of the modified CPS.

To facilitate foliage fertilizing on seedling and tender leaves, an MS system was added to the CPS. The system can be turned on and off independently. This modification lays the basis for comparing the three fertilizing methods. Figures 1 and 2 show the original and modified CPSs, respectively.

### 2.3 Experimental design

The soybean variety used in our experiments is Dongnong 67. The seeding depth, row spacing, and plant spacing were set to be 2.5, 40, and 20 cm, respectively. The base fertilizer is a mixture of diammonium phosphate (N + P_2_O_5_ ≥ 64.0%) and compound fertilizer (N + P_2_O_5_ + K_2_O ≥ 45%) at the ratio of 1:2. During seedling, the base fertilizer was applied uniformly at the rate of 400 kg/hm^2^.

The experiments were carried out from May 9 to September 18, 2018. Three factors were considered in the experiments, namely, amount of water (W), fertilizing rate (N), and fertilizing method (F). Each factor was divided into three levels. Hence, the experiments follow a multifactor orthogonal plan L_9_(3^4^), involving the following combinations: zone 1 (W1N1F1), zone 2 (W1N2F2), zone 3 (W1N3F3), zone 4 (W2N1F2), zone 5 (W2N2F3), zone 6 (W2N3F1), zone 7 (W3N1F3), zone 8 (W3N2F1), and zone 9 (W3N3F2). Each combination was tested three times.

As shown in Table 1, the amount of water was divided into low (W1), medium (W2), and high (W3). The amount of water covers the water used for topdressing in the branching stage (July 1) and in the flowering stage (August 5). As shown in Table 2, the topdressing amounts in the two stages were the same under different combinations.

**Table 1:** Amount of water for soybean in different growth stages

Amount of water	I/mm						
	May 18	June 2	June 8	July 1	July 10	August 5	Total
W1	12.43	12.43	12.43	5	5.33	5	52.62
W2	18.65	18.65	18.65	5	7.46	5	73.41
W3	37.3	37.3	37.3	5	16.7	5	138.6

**Table 2:** Topdressing in different growth stages

Levels of topdressing	Fertilizing rate N/(kg/hm^2^)		
	Type of fertilizer		Topdressing amount
	Monopotassium phosphate	Urea	
N1	1.5	5.25	6.75
N2	2.25	7.5	9.75
N3	3	10.5	13.5

**Figure 3:**
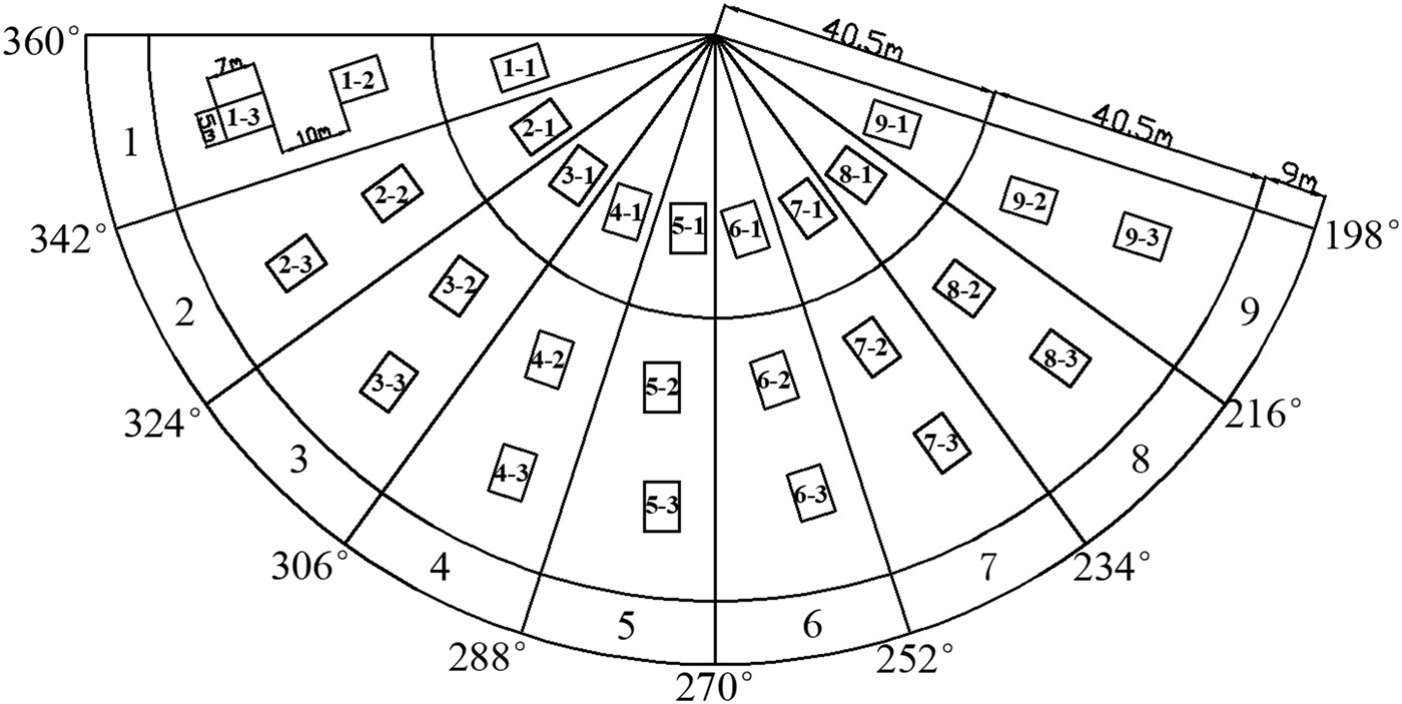
Layout of plots in the CPS coverage.

As mentioned before, three fertilizing methods were compared in our experiments: CPS fertigation (F1), MS fertigation (F2), and MS fertilizing + CPS spraying and leaching (F3). Since each of the nine combinations was tested three times, a total of 27 plots were arranged on the experimental site. The layout of the plots in the coverage of the CPS is given in Figure 3.

### 2.4 Measurement items and methods

#### 2.4.1 Meteorological data

On the experimental site, a meteorological station was set up to monitor the meteorological data in real time, including rainfall, air pressure, temperature, relative humidity, evaporation, wind speed, and ground temperature.

#### 2.4.2 Growth traits

At the end of each growth stage, the plant height was captured using a tape measure, and the stem diameter was measured with a Pro’sKit high-precision digital Vernier caliper. During the measurement, three plots were randomly selected from different zones. Then, three plants were randomly chosen from each plot. Hence, a total of nine plants were identified for measurement. The average height and stem diameter of these plants were taken as the final results.

#### 2.4.3 Yield indices

The yield indices include the total number of seeds per plant, 100-seed weight, and yield. The measurement approach of each index is given in Table 3.

**Table 3:** Yield indices

No.	Indicator	Measurement method
1	Total number of seeds per plant	Select 10 random plants from a 5 cm × 5 cm area in each plot; count the number of pods in each plant; count the number of seeds in each pod; calculate the total number of seeds in each plant; take the average as the total number of seeds per plant in the plot
2	100-seed weight	After threshing, select 100 random seeds and weigh them; repeat this step 3 times and take the average as the 100-seed weight (g)
3	Yield	Dry the threshed seeds, weigh them, and convert the weights to kg/hm^2^

### 2.5 Data processing

Excel 2010 and Data Processing System (DPS) V9.01 were adopted to analyze the experimental data; Excel 2010 and AutoCAD2007 were employed to draw the figures.

## 3 Results and discussion

### 3.1 Climate conditions and the amount of water during the study period

The growth stages of soybean are listed in Table 4. The soybeans were planted on May 9, 2018, and harvested on September 18, 2018. The total growth cycle lasted 132 days.

**Table 4:** Growth stages of soybean

Date	Growth stage	Duration/days
May 9	Seeding	0
May 10–June 18	Seedling	40
June 19–July 6	Branching	18
July 7–August 7	Flowering	32
August 8–August 30	Podding	23
August 31–September 18	Seed filling	19
	Total	132

**Figure 4:**
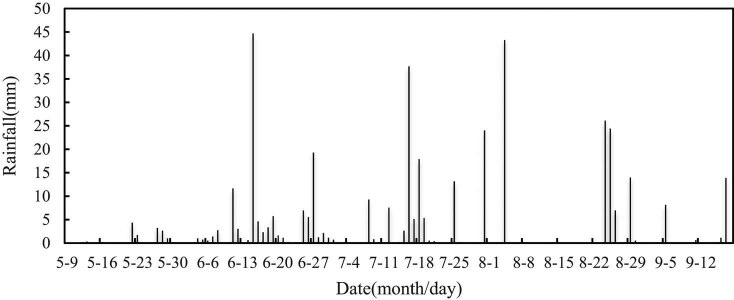
Rainfall in the growth cycle of soybeans.

The rainfall in the growth cycle is illustrated in Figure 4. As can be seen, rainfall mainly occurred in June, July, and August. In the seedling stage, the rainfall was merely 0.5 mm, which is insufficient for the soybeans to germinate normally. This reflects the severe spring drought in Northeast China. The drought suppresses the germination rate, resulting in uneven germination and dead seedlings. After the seedling stage, the rainfall remained too low to support the normal growth of seedlings. At this time, the ground temperature in Northeast China rose sharply, causing serious evaporation of the surface soil moisture. If water is not replenished in a timely manner, soybean seedlings will wither and die due to evapotranspiration.

### 3.2 Effects of different combinations on growth traits

#### 3.2.1 Effects of different treatments on plant height

**Figure 5:**
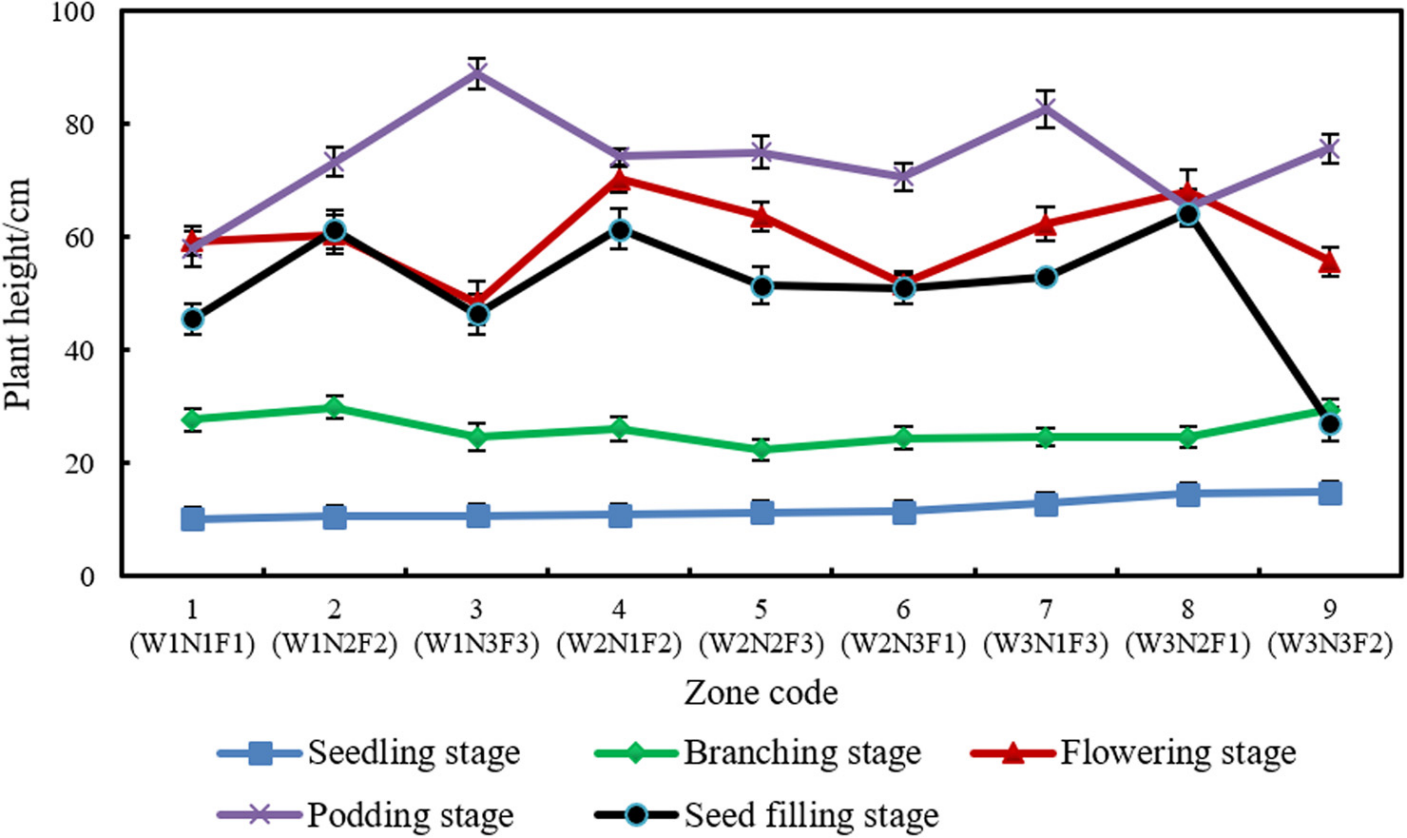
Plant heights in different combinations.

The plant height is an important indicator of the yield and quality of soybean. Figure 5 records the plant heights of the selected plants through different growth stages.

The soybean plants were generally short at the seedling stage. After foliage fertilizer was applied at the branching stage, the plants growth picked up speed. The growth was particularly fast in the flowering stage. Foliage fertilizer was also applied early at the podding stage. After this, the plants grew continuously to the maximum height. Upon entering the seed-filling stage, the plants became shorter, for the nutrients in the stem began to move to the seeds. The soybean stems started to wither and die.

At the seedling stage, amount of water had a significant effect on plant height. The tallest plant (14.8 cm) and the shortest plant (9.1 cm) were from zone 9 (W3N3F2) and zone 1 (W1N1F1), respectively. This means a high amount of water at the seedling stage promotes soybean growth, while a low amount of water at this stage inhibits soybean growth. At this stage, the limited rainfall amplifies the importance of irrigation to plant growth. That is why amount of water greatly affects plant height.

At the branching stage, the plant grew taller at a rapid speed. There is no significant difference between the nine plots in plant height, owing to the onset of the rainy season. The sufficient rainfall fully satisfies the water demand for plant growth. As a result, the soybean plants are similar in height, despite the difference in the amount of water. Even if the plants are irrigated with a low amount of water in the rainy season, the soybean yield will be as high as that of plants fully irrigated [13].

The height of soybean plants is also promoted by fertigation, which provides the nutrients that the plants cannot absorb from the soil in the early phase. For example, the plants are unable to convert nitrogen in the air into nitrogen-containing substances that plants can absorb, before the formation of rhizobia. To ensure normal growth, it is necessary to supplement nutrients in a timely manner.

The soybean plants in our experiments were cropped continuously for two consecutive years, causing a serious lack of nutrients in the soil. To make up for this shortage, foliage fertilizer should be applied in time. Our results show that timely foliage fertilizing protected normal plant growth. Thus, it is recommended to apply foliage fertilizer timely for continuously cropped soybeans.

#### 3.2.2 Effects of different combinations on stem diameter

**Figure 6:**
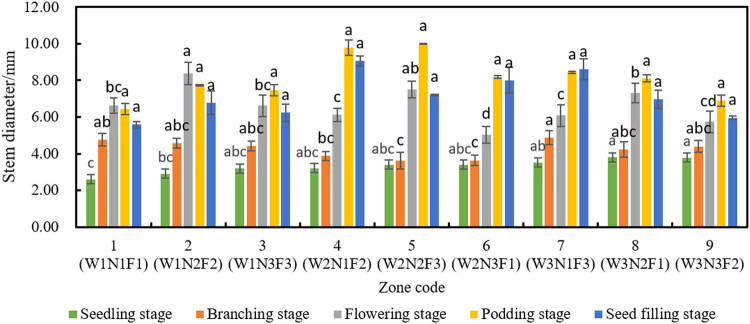
Stem diameters in different combinations. According to the design of orthogonal experiments, the variances were calculated on DPS v.9.01; the lowercase letters indicate that the variables at the same stage have significant differences at the *p* < 0.05 level.

The stem channels water and nutrients to every part of the plant. The thicker the stem, the better the growth of soybean plant. Figure 6 records the stem diameters of the selected plants through different growth stages.

As shown in Figure 6, amount of water (W) had a significant effect on stem diameter at the seedling stage. The three levels of amount of water can be ranked as high amount > medium amount > low amount in the descending order of stem diameter. The thickest stem (3.8 mm) and the thinnest stem (2.6 mm) appeared in zone 8 and zone 1, respectively.

At the branching stage, the stem diameter under the low amount of water showed the most significant growth (57.69%) from that at the seedling stage, while those under the medium and high amounts of water showed less significant yet large growths (10.89% and 21.78%, respectively). This is attributable to the rainfall increase in the later stage and the irrigation replenishment in the early stage. At the branching stage, the fastest growth belongs to the stem diameter of soybean plants irrigated with a low amount of water, indicating that the irrigation quota of soybean needs to be adjusted timely according to rainfall [14].

At the flowering stage, stem diameter was greatly affected by the amount of water and fertilizing rate. By stem diameter, the three levels of the amount of water can be ranked as low amount > medium amount > high amount. The reason is that rainfall is sufficient to meet the water demand of soybean growth, after the onset of the rainy season. At this time, the stem diameter under a low amount of water was much greater than those under medium and high amounts. Under the latter two conditions, there was no significant difference in stem diameter.

Similarly, the three levels of fertilizing rates can be ranked as medium rate > low rate > high rate. This means proper fertilization helps soybean stem grow thicker at the flowering stage, while insufficient or excessive fertilization is unfavorable to stem growth. Between low and high rates of fertilization, the soybean plants had no significant difference in stem diameter. On average, the stems under low rate were slightly thicker than those under high rate. Hence, excessive fertilizing rate at the flowering stage inhibits stem growth and should be eliminated.

At the podding stage, the plants from different plots differed slightly in stem diameter. None of the tested factors showed a significant effect on stem diameter. The thickest (8.45 mm) and thinnest (6.44 mm) stems were observed in zone 7 and zone 1, respectively.

At the seed-filling stage, the stem diameters in all plots were smaller than those at the podding stage, due to the nutrient transfer from the stem to seeds. At this time, the stems began to wither and decay. Since the plants from different plots had similar stem diameters, the effects of amount of water and fertilizing rate on stem diameter were strong in the early stage but weak in the middle and later stages.

#### 3.2.3 Effects of different combinations on root length

Crops with a large root system can absorb a large amount of nutrients from a wide range of soil [15]. The roots of soybean have the ability to fix nitrogen. In the middle and later stages of growth, the emerging nodules on soybean roots could absorb nitrogen (N^2^) from the air trapped in the soil and convert it into ammonia (NH_3_). Nearly 70% of the nitrogen required for soybean growth is obtained in this manner [16]. Therefore, root length and density reflect the nitrogen-fixation capacity and growth condition of soybean plants.

**Figure 7:**
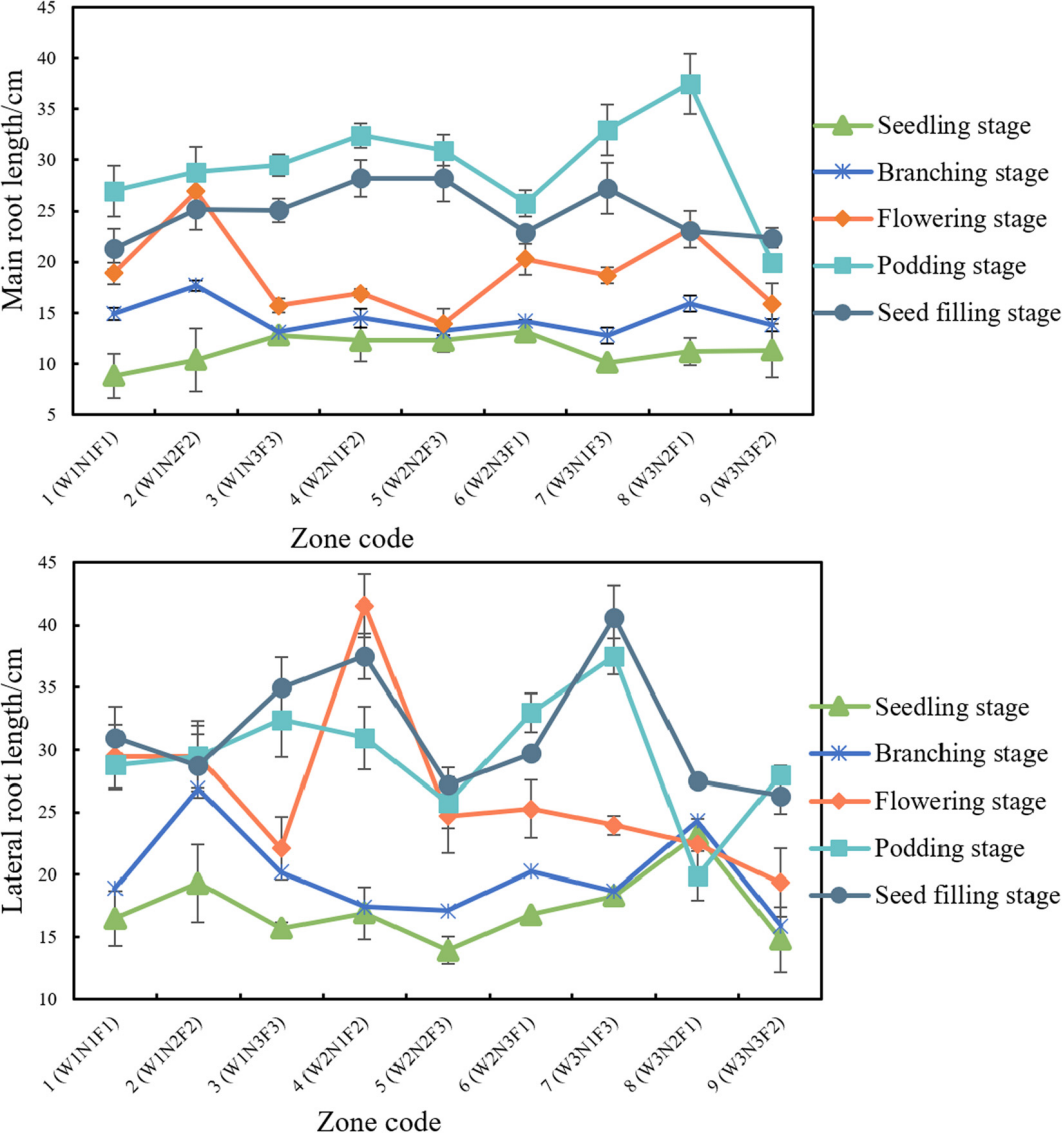
Main root lengths and lateral root lengths in different combinations.

The roots of soybean can be divided into main root and lateral roots. The main root refers to the root that grows right after germination, while lateral roots refer to the roots branched from the main root. In our experiments, soybean plants were sampled randomly from different plots at different growth stages. Then, the lengths of the main root and longest lateral root were measured for each sample. The results are displayed in Figure 7.

As shown in Figure 7, the main root length minimized at the seedling stage, increased rapidly thereafter, and peaked at the podding stage. The peak was followed by a gentle decline, that is, the main root was shrinking. The reason is that at the seed filling stage, the soybean ceases to grow, and the nutrients migrate from stems and leaves to the seeds. This is when the soybean roots end their mission and start to wither and die.

Branched from the main root, lateral roots are generally longer than the main root. Their mission is to absorb more water and nutrients from a wider range. Our experimental results show that similar to the main root, the lateral roots were the shortest at the seedling stage. During the flowering stage, the lateral roots extended rapidly. Into the podding stage, they continued to grow. The growth trend persisted even into the seed-filling stage, probably because lateral roots decay later than the main root. After the soybean matures, the lateral roots will remain active to some extent, so that the soybean can absorb water and nutrients from the soil.

Significance analysis shows that none of the tested factors significantly affected the main root length or lateral root length.

### 3.3 Effects of different combinations on yield indices

#### 3.3.1 Effects of different combinations on the total number of seeds per plant

**Figure 8:**
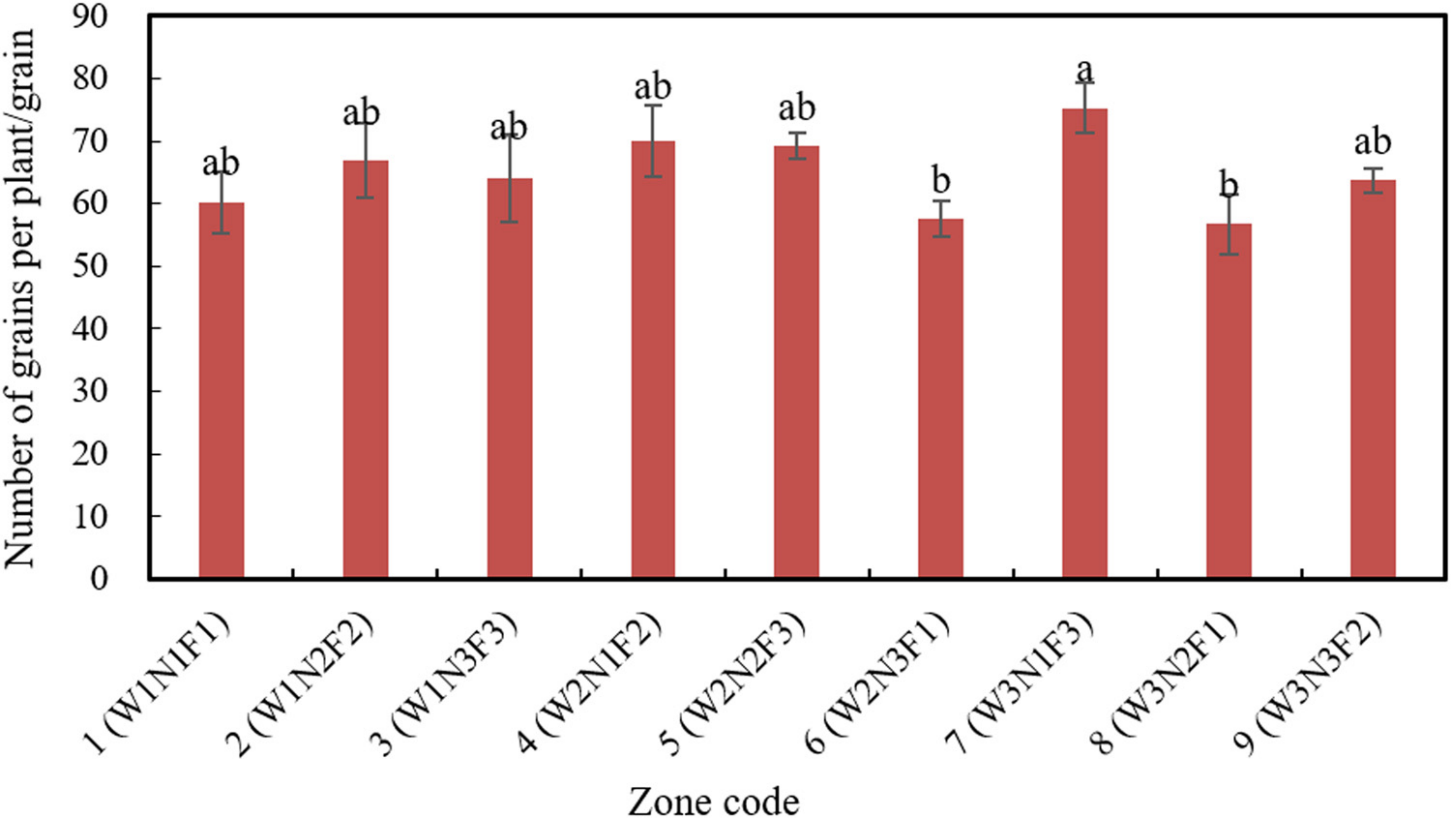
The number of seeds per plant in different combinations. Different lowercase letters in the histogram indicate significant differences at the *P* < 0.05 level.

Figure 8 presents the number of seeds per plant in each zone for different combinations.

In zones 1–9, both amount of water and fertilizing rate exerted insignificant effects on the total number of seeds per plant. The fertilizing method, however, had a significant effect on the total number of seeds per plant: F3 > F2 > F1. Major difference was observed between F3 and F1, but not between F3 and F2 or between F2 and F1.

The maximum total number of seeds per plant (75.3) appeared in zone 7 (W3N1F3), followed by that (70.03) in zone 4 (W2N1F2). The lowest total number of seeds per plant (56.77) appeared in zone 8 (W3NN2F1), and the second lowest number (57.6) appeared in zone 6 (W2N3F1). Therefore, a reasonable fertilizing method improves the nutrient absorption of soybean plants, making the seeds plumper and fertilizing more efficient.

Thanks to its small spray flow and high degree of atomization, the newly added MS system, which applied foliage fertilizer during the growth cycle, improves the fertilizer absorption and utilization of soybean leaves and stems. Since soybean requires less fertilizer in topdressing than other plants, MS fertigation is an excellent choice for soybean to improve the absorption of fertilizer and reduce waste.

#### 3.3.2 Effects of different combinations on 100-seed weight

**Figure 9:**
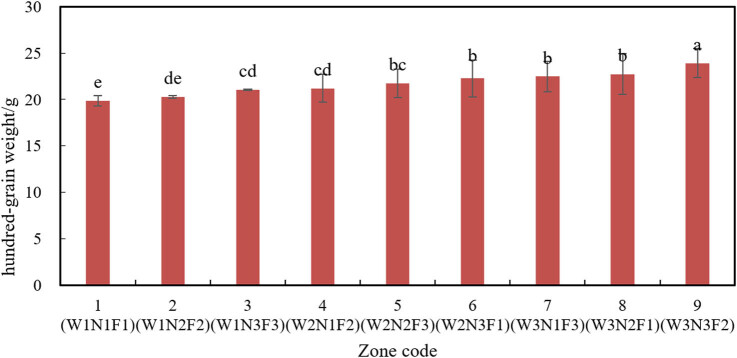
100-seed weights in different combinations.

As presented in Figure 9, variance analysis shows that amount of water (W) and fertilizing rate (N) exerted significantly different impacts on the 100-seed weight of soybean.

In terms of the effect on 100-seed weight, the three levels of W can be ranked as high amount > medium amount > low amount. This is consistent with the common saying that soybean grows better in wet climate, while grains grow better in dry climate. In other words, abundant water is necessary for high soybean yield.

The three levels of fertilizing rate can be ranked as high rate > medium rate > low rate by the effect on 100-seed weight. Hence, more topdressing in the later stages of soybean makes seeds plumper and the 100-seed weight higher, thereby boosting the soybean yield.

The largest 100-seed weight of soybean (23.9 g) appeared in zone 9 (W3N3F2), followed by that (22.73 g) in zone 8 (W3N2F1). The lowest 100-seed weight of soybean (19.87 g) appeared in zone 1 (W1N1F1), and the second lowest (20.27 g) appeared in zone 2 (W1N2F2). Therefore, proper irrigation and fertilization during the growth cycle make the seeds plumper and increase the 100-seed weight of soybean.

#### 3.3.3 Effects of different combinations on yield

**Figure 10:**
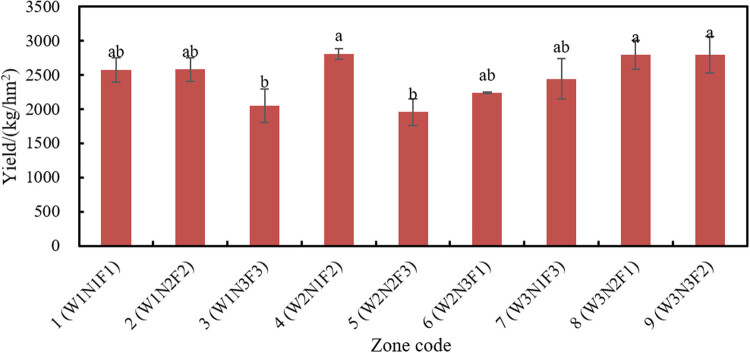
Yields in different combinations.

As shown in Figure 10, the maximum yield (2811.88 kg/hm^2^) appeared in zone 4 (W2N1F2), followed by that (2797.59kg/hm^2^) in zone 9 (W3N3F2); the lowest yield (1960.03 kg/hm^2^) appeared in zone 5 (W2N2F3), and the second lowest yield (2054.45 kg/hm^2^) was observed in zone 3 (W1N3F3).

Neither amount of water nor fertilizing rate had a significant effect on the yield of soybean, while the fertilizing method had a significant effect: F2 > F1 > F3. This means the MS fertigation can greatly bolster the soybean yield, for which fertilizing method is suitable for spraying foliage fertilizer onto soybean. Compared with CPS fertigation, MS fertigation has a low irrigation intensity and a small flow, which protect seedlings and tender leaves and facilitate nutrient absorption. Therefore, it is recommended to adopt the MS fertigation for soybean topdressing.

## 4 Conclusions

(1) At the seedling stage, the amount of water had significantly different effects on plant height, stem diameter, and root length of soybean. With the onset of the rainy season, the amount of water no longer had a significant effect on the growth straits of soybean in the later stage of growth. The farmers in Northeast China are advised to irrigate more water in the early growth stage and less or even no water in the middle and later stages.
(2) In our experiments, foliage fertilizer was sprayed during the branching and flowering stages. The application effectively curbed the yield reduction induced by continuous cropping. Stem diameter was greatly affected by fertilizing rate. The three levels of fertilizing rates can be ranked as medium rate > low rate > high rate based on the effect on stem diameter. Thus, proper fertilization during the flowering stage helps soybean stems grow thicker.
(3) The fertilizing method (F) had a significant effect on soybean yield: F2 > F1 > F3 in terms of effect. This means that the MS fertilization can significantly boost the yield of soybean. Therefore, it is recommended to adopt MS fertigation for soybean topdressing.
(4) The maximum yield (2811.88 kg/hm^2^) was achieved in zone 4 (W2N1F2), followed by that (2797.59kg/hm^2^) in zone 9 (W3N3F2); the lowest yield (1960.03 kg/hm^2^) was achieved in zone 5 (W2N2F3), and the second lowest yield (2054.45 kg/hm^2^) in zone 3 (W1N3F3).
(5) To prevent yield loss, it is necessary to fertigate the soybean at a proper rate by the suitable fertilizing method at the right time in the growth cycle. The farmers are suggested to replace continuous cropping with crop rotation. If continuous cropping is unavoidable, foliage fertilizer should be sprayed timely for topdressing at the flowering and seed-filling stages.
